# Supplementary material for: Predictors for self-discontinuation of anti-osteoporosis medication: A hospital-based real-world study
Source: PLoS One. 2022 Sep 21;17(9):e0275020. doi: 10.1371/journal.pone.0275020 (PMC9491539; doi:10.1371/journal.pone.0275020)
Supplement: Attachment — Submitted filename: rebuttal letter.docx [file pone.0275020.s001.docx]

**Author’s Response to reviewer’s comments and suggestions**

**Reviewer #1**

Point 1: lines 8-10: "Comparisons among groups and risks of self-discontinuation were analyzed. Among 695 patients, 375 (54%) self-discontinued, 64 (9.2%) discontinued due to physicians’ suggestion, and 256 (36.8%) died." The death may not be considered a cause of discontinued therapy. The authors may explain better the concept.

**Author’s response:** Thank you for the comment. We agree with the reviewer’s opinion that the death may not be considered a cause of self-discontinuation of anti-osteoporosis therapy. Mortality should be considered as unintended discontinuation of treatment. Patients enrolled in the unintended discontinuation group mainly included patients with good adherence to anti-osteoporotic medication before death, and they stopped using anti-osteoporotic medication due to mortality. To avoid confusion, we have revised the aim of our study as “to investigate the causes of anti-osteoporosis medication discontinuation and to explore risk factors for self-discontinuation” (Please see page 5, lines 15-17). We also revised Table 2 and Figure 1 to re-categorize the study population.

Point 2: paragraph 2.2: the authors considered a cause of discontinuation of medication the death. This event can't be compared with the others two causes (self-discontinuation and discontinuation due to the physician’s recommendation) if the scope of this study is "to investigate risk factors and causes of poor medication adherence in patients with osteoporosis, with a view to improving medication persistence." Please expose better the concept.

**Author’s response:** Thank you for the suggestion. We agree with the reviewer’s comment that the death can’t be compared with the others two causes if the scope of this study is "to investigate risk factors and causes of poor medication adherence in patients with osteoporosis. We believed that mortality contributes to medication discontinuation and should be classified as unintended discontinuation. As mentioned in the previous reply to reviewer’s comment, we have revised the aim of our study, and re-classify this group to “unintended discontinuation” (Please see page 7, lines 13-14, Table 2 and Figure 1).

**Reviewer #2**

First, what were the definitions of discontinuation of all the medication except for the denosumab and teriparatide mentioned in the method part? Do you think it is more reasonable to set up the definition of discontinuation according to the half-life of each of the selected medication? Or, please state your criteria accordingly.

**Author’s response:** Thank you for bringing up this critical comment. We have added the definition of bisphosphonate or raloxifene discontinuation as ≥12 months without any anti-osteoporosis medication prescription claims after prior bisphosphonate or raloxifene treatment (please see page 6, line 26; page 7, lines 1-2). We agree with the reviewer’s opinion that it seems to be reasonable to set up the definition of discontinuation according to the half-life of each of the selected medication. However, the dosing frequency may last beyond the half-life of each medication in blood, i.e., biologic effect might be different from pharmacokinetics of medication. For example, the half-life of denosumab is less than 30 days but the dosing frequency is every 6 months. Therefore, we defined the duration of discontinuation of each anti-osteoporosis medication according to previous publications [1-5].

Reference

1.	Silverman SL, Siris E, Kendler DL, Belazi D, Brown JP, Gold DT, et al. Persistence at 12 months with denosumab in postmenopausal women with osteoporosis: interim results from a prospective observational study. Osteoporos Int. 2015;26(1):361-72. Epub 2014/09/23. doi: 10.1007/s00198-014-2871-6. PubMed PMID: 25236877; PubMed Central PMCID: PMCPMC4286624.

2.	Migliaccio S, Francomano D, Romagnoli E, Marocco C, Fornari R, Resmini G, et al. Persistence with denosumab therapy in women affected by osteoporosis with fragility fractures: a multicenter observational real practice study in Italy. J Endocrinol Invest. 2017;40(12):1321-6. doi: 10.1007/s40618-017-0701-3. PubMed PMID: 28589380.

3.	Burge R, Sato M, Sugihara T. Real-world clinical and economic outcomes for daily teriparatide patients in Japan. Journal of bone and mineral metabolism. 2016;34(6):692-702. Epub 2016/11/01. doi: 10.1007/s00774-015-0720-0. PubMed PMID: 26661475.

4.	Chen Q, Guo M, Ma X, Pu Y, Long Y, Xu Y. Adherence to Teriparatide Treatment and Risk of Fracture: A Systematic Review and Meta-Analysis. Horm Metab Res. 2019;51(12):785-91. doi: 10.1055/a-1062-9447. PubMed PMID: 31826274.

5.	Adami G, Jaleel A, Curtis JR, Delzell E, Chen R, Yun H, et al. Temporal Trends and Factors Associated with Bisphosphonate Discontinuation and Restart. J Bone Miner Res. 2020;35(3):478-87. doi: 10.1002/jbmr.3915. PubMed PMID: 31714637; PubMed Central PMCID: PMCPMC7401723.

Second, why the discontinuation rate of denosumab is the highest among all the medications, which is contrary to our clinical experiences, especially compared to those short half-life oral medications? Is it due to the definition of inclusion criteria or selection bias?

**Author’s response:** Thank you for the comment. In our study, patients discontinuing denosumab accounts for the majority among 3 groups of different discontinuation causes (Table 2). It could be resulted both from the inclusion criteria and selection bias. We enrolled participants who discontinued anti-osteoporosis medication between June 2016 and June 2018. Because the denominators of the discontinuation rates were not calculated, the highest proportion that denosumab accounted for might be due to the largest market share of it. However, our retrospective study design prevented us from comparing discontinuation rates among various anti-osteoporosis medication. We discussed this point in the limitation section (please see page 17, lines 9-12).
